# The Embryonic Septum and Ventral Pallium, New Sources of Olfactory Cortex Cells

**DOI:** 10.1371/journal.pone.0044716

**Published:** 2012-09-11

**Authors:** María Laura Ceci, María Pedraza, Juan A. de Carlos

**Affiliations:** 1 University of Chile, Las Encinas, Santiago, Chile; 2 Department of Molecular, Cellular and Developmental Neurobiology, Instituto Cajal- Consejo Superior de Investigaciones Científicas, Madrid, Spain; University of Salamanca- Institute for Neuroscience of Castille and Leon and Medical School, Spain

## Abstract

The mammalian olfactory cortex is a complex structure located along the rostro-caudal extension of the ventrolateral prosencephalon, which is divided into several anatomically and functionally distinct areas: the anterior olfactory nucleus, piriform cortex, olfactory tubercle, amygdaloid olfactory nuclei, and the more caudal entorhinal cortex. Multiple forebrain progenitor domains contribute to the cellular diversity of the olfactory cortex, which is invaded simultaneously by cells originating in distinct germinal areas in the dorsal and ventral forebrain. Using a combination of dye labeling techniques, we identified two novel areas that contribute cells to the developing olfactory cortices, the septum and the ventral pallium, from which cells migrate along a radial and then a tangential path. We characterized these cell populations by comparing their expression of calretinin, calbindin, reelin and Tbr1 with that of other olfactory cell populations.

## Introduction

Migration is an essential process in the development of the nervous system, allowing newly generated cells to reach sites far from their origin. Indeed, all nervous system structures represent aggregates of distinct cell populations that are actually generated in distinct areas, each expressing different cell markers and using specific migratory pathways to reach their final target destinations. Two main migratory mechanisms have been described in the nervous system: radial and tangential migration (for review see [1]). While the former is dependent on radial glia cells and generally involves cell migration over short distances [2], tangential migration occurs independently of glia cells and it is more widespread in earlier developmental stages, before glial cell maturation [3]–[5]. These migratory pathways can overcome barriers between different developmental vesicles, with no limit to the distances over which cells can migrate.

The sources of cells that form specific encephalic structures are often investigated using tissue slices that are labeled and cultured in plates, whereas in the present study we have analyzed cell migration during development in whole embryos. This approach ensures that rostro-caudal migratory routes develop normally, thereby circumventing several drawbacks of the tissue slice approach, including the loss of targets and cell guidance cues and the erroneous migration of newly generated cells.

We previously demonstrated that olfactory cortex cells are generated in most telencephalic proliferative areas just before the cortical preplate is split by the accumulation of newly generated cortical plate cells [4]. In the present study, we focused on two proliferative areas not featured in our previous analyses, the septal area and the ventral pallium (the pallial region located next to the pallium-subpallium boundary). Using fluorescent tracer injections, we characterized the migratory pathways of cells of septal and ventral pallial origin at different developmental stages (E10.5 to E12.5) to determine the individual contributions of these proliferative regions to the developing brain. Moreover, we investigated whether these regions give rise to olfactory cortex cells during this developmental window, or to more heterogeneous populations that can also reach the cerebral cortex, as proposed by other authors [6]–[8].

## Results

### Cell Migration from the Septum

**Figure 1:**
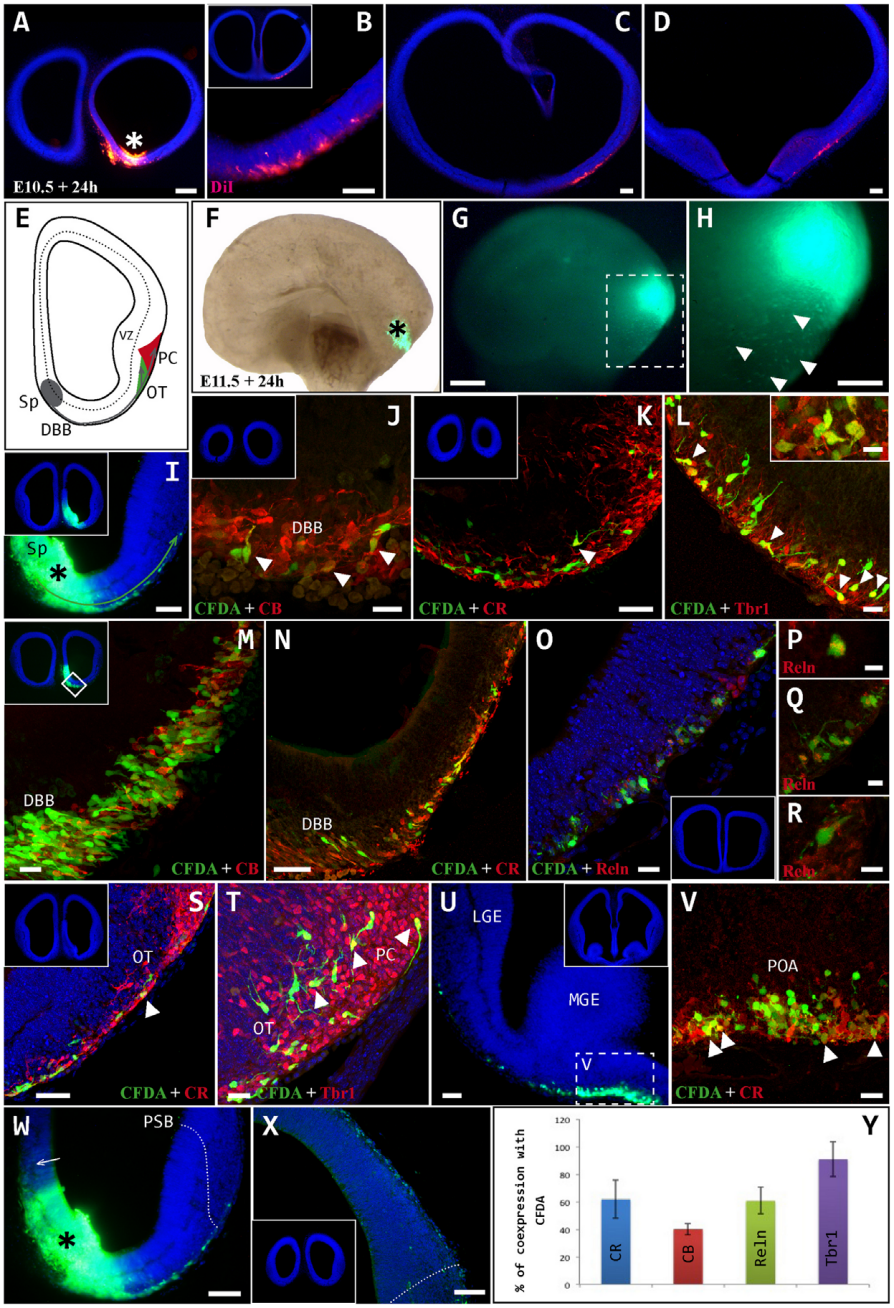
Neuronal migration from the septum. A–D: A DiI crystal was inserted *exo-utero* into the septum (asterisk in A) of an E10.5 embryo, which was then cultured in roller bottles for 24 hours. At this stage, migration from the septum was minimal and the cells migrated tangentially through the outer layer of subpallium in a caudal direction to reach the medial regions of the antero-posterior axis (B–D). E–Y: CFDA tracer was injected *exo-utero* into the septum (asterisks in F, I, G and H) of an E11.5 embryo, which was then cultured in roller bottles for 24 hours. E: Cartoon representing two-dimensional cell migration from the septum (Sp) towards the developing olfactory regions, the olfactory tubercle (OT) and piriform cortex (PC). F, G: Lateral view of the whole brain following CFDA injection into the sp (asterisk), bright (F) and dark field (G) views. H: Enlarged image of box in G, showing the displacement of labeled cells through the telencephalon in three dimensions (arrowheads). I–T: From the injection site (I, asterisk), cells migrated radially to the outermost layer, and from there they migrated ventrally and caudally to pass through the diagonal band of Broca (DBB) on the way to the olfactory cortex. On reaching this structure, the cells occupied the interior part of the olfactory tubercle (OT) and the superficial region of the piriform cortex (PC). U–V: Some caudally migrating cells invaded the region corresponding to the preoptic area (POA). W–X: Cells generated in the Sp (asterisk) did not ascend along the medial wall (arrow) or cross the pallial-subpallial boundary (PSB) to enter in the cortical neuroepithelium. Y: Percentage of cells expressing each marker: CR (62%), Tbr1 (91%), CB (40%) and Reln (61%). Arrowheads indicate CFDA-labeled cells co-expressing different markers. Coronal sections: midline is to the left and dorsal is up. Blue cells are counterstained with bisbenzimide. Scale bars: A, F, G: 200 µm; B-D, H, I, W, X: 100 µm; K, N, S, U: 50 µm; J, L, M, O, T, V: 20 µm. Boxes in L, Q, R: 10 µm; P: 5 µm.

To determine the fate of cells generated in the septal area at E10.5 and E11.5, fluorescent tracers (CFDA or DiI) were injected *exo utero* into whole embryos that were then cultured in roller bottles for 24 hours. Embryos injected at E10.5 exhibited very few cells of septal origin (Fig. 1 A), which initially followed a ventral migratory pathway and subsequently switched to a lateral pathway to move away from the midline through the outermost subpallial layer. The cells then migrated caudally to ultimately reach the mid-region of the telencephalon in the rostro-caudal axis (Fig. 1 A–D). One day after septal injections in E11.5 embryos, large scale migration of labeled cells was observed along ventral and lateral routes in the direction of the olfactory cortex (Fig. 1 E-H). The cells initially migrated radially from the septal ventricular zone to the outermost layer, subsequently migrating tangentially through the diagonal band of Broca (DBB) to reach the olfactory tubercle and piriform cortex (Fig. 1 I–T). These cells exhibited complex migratory behavior through the three dimensions of the telencephalon. Some cells migrated in clusters, occupying a tissue thickness of approximately 40 µm (Fig. 1 K–L). In the olfactory cortex, we observed a variety of cell morphologies, including fusiform cells with single processes and bipolar cells (Fig. 1 T), indicating that cell differentiation begins very early in the olfactory area. In addition to invading the olfactory cortex, the migrating cells also reached the preoptic area (Fig. 1 U, V), although they did not invade the neocortex and failed to cross the dorsal limit of the piriform cortex (subpallium to pallium), or to follow a dorsal migratory route (via the medial wall: Fig. 1 W, X).

**Figure 2:**
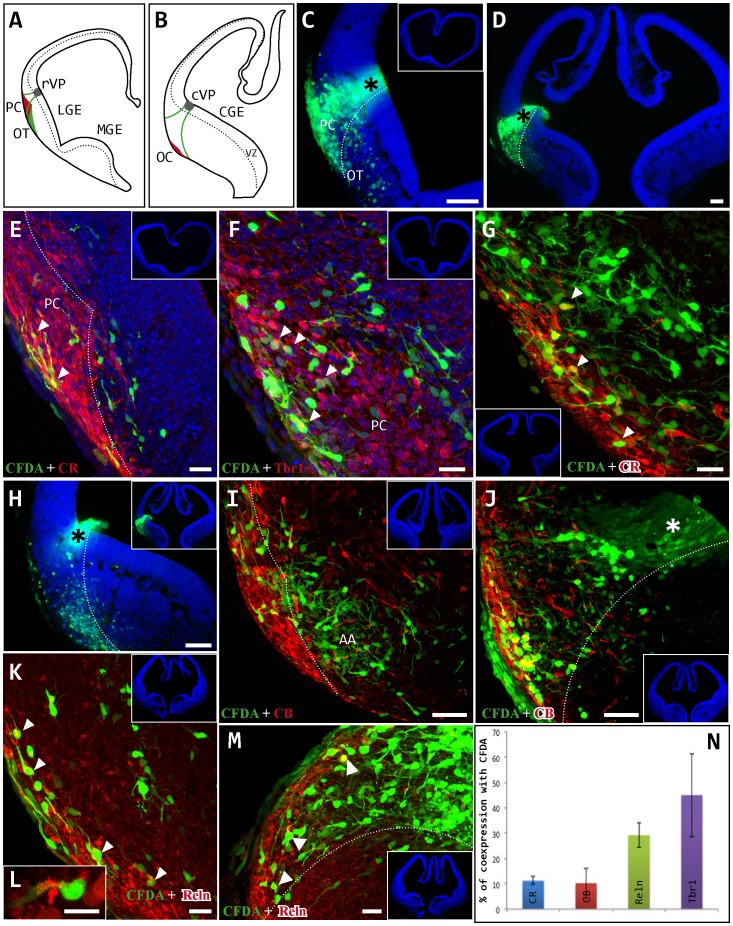
Neuronal migration from the ventral pallium. CFDA tracer was injected *exo-utero* into E11.5 embryos that were then cultured in roller bottles for 24 hours. A, B: Schematic drawings showing the location of the injection site (grey circle) in the rostral ventral pallium (rVP) and the caudal ventral pallium (cVP). C, D: Labeled cells migrated from their origin (black asterisk) towards the pial surface through the mantle of the lateral ganglionic eminence (LGE) to reach the piriform cortex (PC). Few cells invaded the area corresponding to the olfactory tubercle (OT). Dotted lines define the boundary between the PC and OT. E–G: Embryo injected in the rVP. CFDA-labeled cells (green) occupied the rostro-caudal extension of the PC. Images show immunohistochemistry (red cells) for calretinin (CR; E, G) and Tbr1 (F), and arrowheads indicate double-labeled cells. H-M: Embryo injected in the cVP (asterisk). CFDA-labeled cells (green) migrated along the rostro-caudal axis of the brain to occupy almost the entire length of the olfactory cortex (OC), even invading amygdaloid areas (AA; I). These cells were not restricted to the most superficial portion but also populated the depth of the PC (J–M). Images show immunohistochemistry (red cells) for calbindin (CB; I, J) and reelin (Reln; K–M). N: Percentage of cells expressing each marker: CR (11%), Tbr1 (45%), CB (10%) and Reln (29%). Coronal sections; midline is to the right and dorsal is up. Blue cells are counterstained with bisbenzimide. Scale bars: C–E, H: 100 µm; I, J: 50 µm; E–G, K-M: 20 µm.

By combining immunohistochemistry with lineage tracing in whole embryos, we characterized the diverse cell populations originating in the septum. In general, these populations were strongly immunoreactive for calretinin (CR), Tbr1, calbindin (CB) and reelin (Reln: Fig. 1 Y). Almost all cells expressed the pallial marker Tbr1 (91%), many expressing CR (62%) and Reln (61%). Moreover, 40% expressed CB, as is typical of subpallial populations.

### Cell Migration from the Ventral Pallium

The migratory behavior of the cell populations arising from the ventral pallium was analyzed by injecting the CFDA fluorescent tracer into whole E10.5/11.5 embryos that were subsequently cultured in roller bottles. The ventral pallium occupies the more dorsal aspect of the lateral ganglionic eminence (LGE) and it is delimited ventrally by the pallial-subpallial border. As observed in the septum, the contribution of ventral pallium cells to other areas at E10.5 was limited (data not shown). However, at E11.5 we observed significant migration of cells from this region, initially following a radial migration route from their site of origin in the rostral and caudal-ventral pallium (rVP, cVP) to reach the marginal zone of this structure (Fig. 2 A–D). These cells subsequently altered their migratory route and their mechanism of displacement to travel tangentially in a rostro-caudal direction through the piriform cortex. Thus, cells generated in the ventral pallium of E11.5 embryos migrated tangentially to occupy areas up to the caudal portion of the piriform cortex (Fig. 2 E–M) and the medial amygdaloid nuclei (AA: Fig. 2 I). Finally, these cell populations occupied the entire extent of the piriform cortex, both rostro-caudally and in the deeper mantle zone. Interestingly, these migratory pathways were highly specific, with few cells crossing the border between the piriform cortex and olfactory tubercle (Fig. 2 C, D, H, J, M).

**Figure 3:**
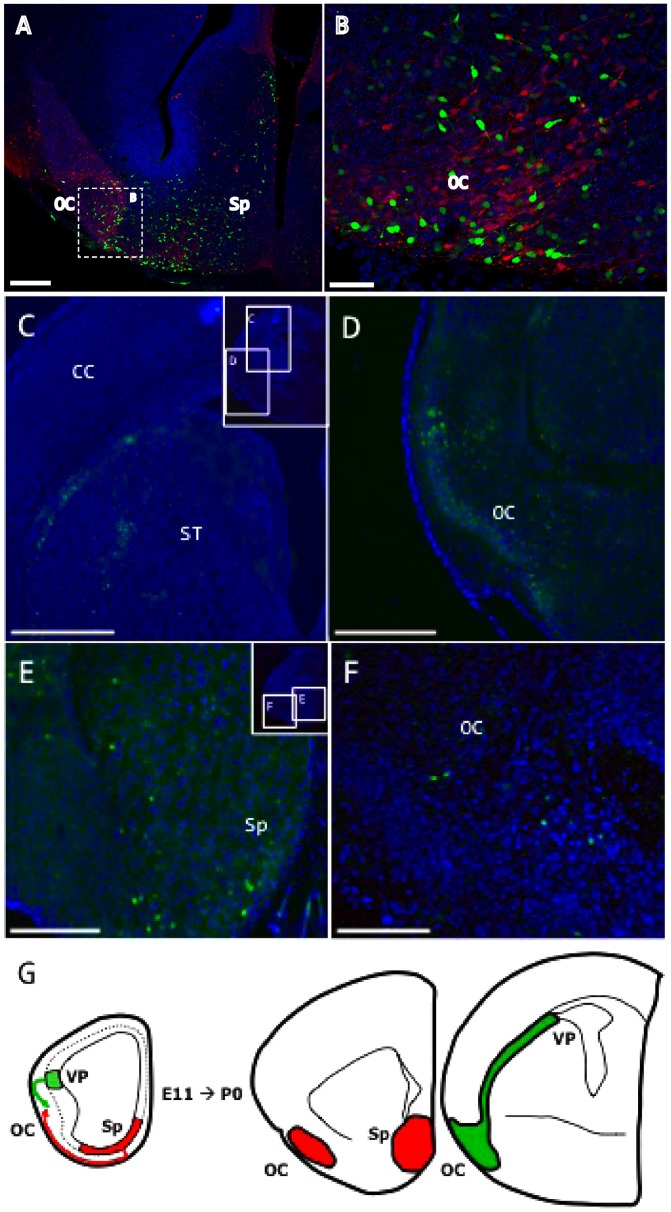
Neuronal migration from the ventral pallium and septum from E11.5-E15.5 (A–B) and E11.5-P0 (C–F). CFDA fluorescent tracer was injected *in utero* at E11.5 using an ultrasound guided system. A–B: CFDA-labeled cells (in green) generated at E11.5 in the septum (Sp) and analyzed 4 days later. CB expression is shown in red. In E15.5 embryos, cells generated in the septal area failed to reach the cerebral cortex (CC). C–D: CFDA-labeled cells (green) generated at E11.5 in the ventral pallium (VP) and analyzed at P0. In neonatal mice, VP cells migrated toward the olfactory cortex (OC) and did not invade the cerebral cortex (CC). E–F: Labeled cells (green) generated in the septum (Sp) at E11.5 only colonized the OC during embryonic development and failed to occupy the cerebral cortex. G: Schematic drawings showing the migratory routes of cell populations generated in the VP and Sp from E11.5 to P0, and the localization of these cells at their target destinations. Coronal sections; midline is to the right and dorsal is up. The tissue was counterstained with bisbenzimide (blue). Scale bars: A, E, F: 100 µm; C, D: 500 µm; B: 200 µm.

**Figure 4:**
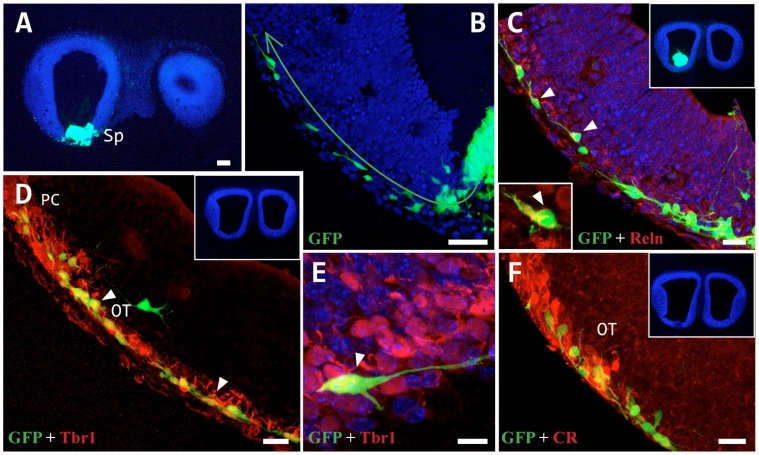
Neuronal migration from a cortical hem tissue grafted into the septum. A: Caudal cortical hem tissue from a GFP^+^ mouse embryo (E11.5) grafted into the septum of a wt mouse (E11.5), which was then cultured for 24 h *in toto*. B–F: GFP^+^ cells reached the outermost layer and migrated tangentially along a ventral route to the superficial layer of the olfactory tubercle (OT) and the piriform cortex (PC). The migrating cells expressed markers typical of Cajal-Retzius cells (arrowheads), such as Reln (C), Tbr1 (D, E) and CR (F), but not CB. Scale bars: A: 100 µm; B, D: 50 µm; C, F: 20 µm; E: 10 µm.

Cell populations migrating from the ventral pallium were characterized by their morphological heterogeneity, preferentially displaying a fusiform and bipolar morphology oriented in different planes. Individual markers were expressed by the migrating cells as follows: CR, 11%; Tbr1, 45%; CB, 10%; and Reln, 29%. We have not found markers presenting a high percentage of co-expression with the CFDA population (Fig. 2 N).

### Cell Migration from the Septum and Ventral Pallium: Postnatal Analyses

We next injected the CFDA tracer *in utero* into E10.5/11.5 embryos using an ultrasound-guided system (VeVo 770®) to confirm that our experimental design allowed sufficient time for cells of the septum and ventral pallium to reach their respective adult destinations, and to rule out the possibility that these cells migrate to areas such as the cerebral cortex, as previously proposed [6]–[8]. Analysis of the resulting labeling in embryonic (E15.5; Fig. 3 A–B) and neonatal (P0; Fig. 3 C–F) mice revealed comparable migratory patterns, whereby cells generated in the septum settled in the olfactory cortex (piriform cortex and olfactory tubercle) and failed to reach the cortical mantle. The marker CB (red in Fig. 3 A–C) was used to identify the olfactory population. Similarly, *in utero* tracer injections into the ventral pallium revealed comparable patterns in E15.5 and E11.5 embryos, with cells spreading throughout the piriform cortex, indicating that no CFDA-neurons, labeled from the VP, colonize the neocortex (Fig. 3 C, D, G).

*In vitro* and *in vivo* fluorescent tracer experiments in whole embryos revealed no migration from the septum and ventral pallium to the cerebral cortex, with labeled cells detected exclusively in the olfactory cortex. As these findings contrast with some accounts of the generation of Cajal-Retzius cells in these areas [6]–[8] (the first cells to populate the neuroepithelium, occupying the outer-most part of this stratum [9], [10]), we next investigated whether the subpallial environment permits tangential migration of this cell type to neocortical structures. After grafting explants from the cortical hem of GFP-transgenic mice (where Cajal-Retzius cells originate [11], [12]) into the septum of wild type embryos at E11.5, only cell migration to the olfactory cortex was observed (Fig. 4 A, B), following the same migratory pathway observed in our CFDA tracer experiments (Fig. 1). The implanted GFP cells predominantly expressed Reln and Tbr1, typical markers of Cajal-Retzius cells (Fig. 4 C-E), and very low levels of CR (Fig. 4 F). Similar results were obtained with cortical hem explants grafted into the ventral pallium, as reported previously [12].

**Figure 5:**
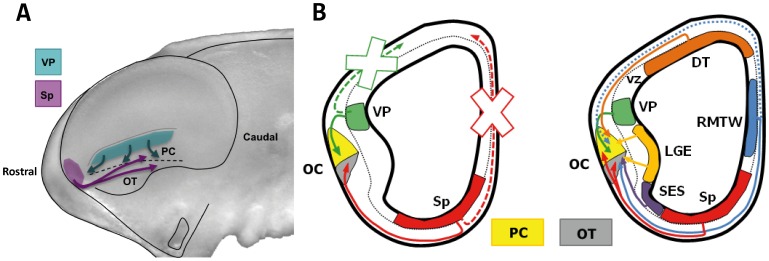
Cartoon summarizing the reported migrations toward the olfactory cortex. A: Cartoon representing the whole embryonic brain showing cell migration from both the Sp and VP. B: Cartoon indicating all areas that give rise to the cells in the olfactory cortex (OC), based on a recent report by our group [11] and including the two new areas identified here: the ventral pallium (VP) and septum (Sp). These are integrated into the scheme on the right and depicted separately on the left. Crosses indicate non-migratory movements to the dorsal telencephalon (DT). Dotted blue line represents a minor migration from the rostromedial telencephalic wall (RMTW). Abbreviations: vz, ventricular zone; LGE, lateral ganglionic eminence; SES, septo-eminential sulcus; PC, piriform cortex; OT, olfactory tubercle.

## Discussion

In the present study, we systematically analyzed the migratory behavior of cell populations generated in the ventral pallium and septal areas using whole embryos, identifying these areas as new sources of olfactory cortex cells in addition to those described previously [4]. Moreover, we demonstrated that cells generated in the ventral pallium and septum during the preplate stage (E10.5–E12.5) do not transgress the pallial-subpalial boundary to enter the cortical neuroepithelium.

### Olfactory Cells Arise from the Ventral Pallium and Septum

The present findings unequivocally demonstrate that cells generated in the septal area invade the olfactory cortex at E11.5, although they fail to reach the medial pallium or cortical preplate. Immunohistochemical characterization of the labeled cells suggests the co-existence of three cell subpopulations: CB^+^/Tbr1^+^ cells (representing 40% of labeled cells), and CR^+^/Tbr1^+^ and Reln^+^/Tbr1^+^ subpopulations that constitute the remaining 60%.

**Table 1:** The distribution of the animals used and their survival.

					Survival >24 h		
		VP	Sp	Total	VP	Sp	Total
	Transplants (E11.5)	8	7	15	**6**	**6**	**12 (75%)**
Inculture	Injections (E10.5)	4	4	8	**4**	**4**	**8 (100%)**
	Injections (E11.5)	17	20	37	**12**	**14**	**26 (71%)**
	Injections (E12.5)	4	4	8	**1**	**2**	**3 (30%)**
Inutero	Injections (E11.5)	5	5	10	**5**	**5**	**10 (100%)**
	Total	38	40	78	28	31	59

Similarly, cells generated at E11.5 in the ventral pallium migrate radially towards the adjacent pial surface to occupy the piriform cortex, from where they spread tangentially and caudally along the antero-posterior axis of the brain to occupy the entire extension of the piriform cortex, but not the olfactory tubercle. Immunohistochemical characterization of the cells originating in the ventral pallium revealed little co-expression of different antibodies with the CFDA tracer, suggesting that at this early developmental stage these cells have not yet differentiated in the mantle of the LGE, and that the cell markers analyzed are only produced in the outermost layer of the olfactory cortex. Most of the differentiated cells expressed Tbr1 and CB, typical markers of the pallial and subpallial regions, respectively, suggesting a mixed lineage at the pallial-subpallial boundary. Indeed, three genetically distinct regions have been previously described in this area [13]. We confirmed our findings by the postnatal analysis of embryos injected with fluorescent tracer using an ultrasound-guided system.

Based on our findings, we can only state that more olfactory cortex cells are generated in the ventral pallium than the septum. This may be due to the different migratory distances associated with each region, although cell contributions from the same area may differ depending on the migratory route taken, as described for the RMTW (rostromedial telencephalic wall [4]) from which a minority dorsal cell population and a majority ventral population migrate to the olfactory cortex. To date, and given to the imprecise nature of the tracing methods used (we can only refer to migratory populations and not to cell number, as each tracing experiment labels different numbers of cells in each area), it is impossible to perform this experiment in a quantifiable manner.

Several authors have proposed that olfactory cells are generated in the pallium and septum. Indeed, we previously described a cell population originating in the dorsal LGE that migrates to the olfactory cortex [3], [4], suggesting that olfactory cell populations are also generated in the pallial zone of this structure, the ventral pallium. Furthermore, a lateral migratory stream has been described [13] that carries cells towards the olfactory cortex and amygdala from the pallial-subpallial border [14], [15], although these analyses were performed at later developmental stages than those of the present study. Finally, grafting of cortical hem explants into the ventral pallium [12] and septum (data presented herein) revealed the existence of tangentially migrating cells that reach the olfactory cortex but not the cerebral mantle.

### The Ventral Pallium and Septum are not Sources of Cortical Neurons

Several studies have proposed the septum and ventral pallium as sources of cortical interneurons [6]–[8], although such studies may be confounded by the difficulties in anatomically discriminating between the septum and preoptic area in embryonic mice. In adults, both these regions are contiguous but occupy distinct areas in the rostro-ventral telencephalic axis, while in embryos these proliferative areas are difficult to distinguish and may be considered a single entity. Accordingly, *in utero* tracer injection into the septal region in E11.5 embryos labels both the septum and preoptic areas, and may lead to the tracing of the cell populations arising from both regions. We demonstrated that these cells fail to enter the cortical mantle, consistent with previous findings [16], at preplate stages (E10.5–E12.5). Although our experiments point to the same results at later stages, we just suggest that cells fail to enter the cortex at these stages, because of the possible lack of the CFDA-labeling after further divisions.

Based on the expression pattern of specific markers [7], [8], several authors have proposed the embryonic preoptic area as a site of cortical interneuron generation, while others have described the migration of both interneurons and transient glutamatergic cells from the ventral pallium to the neocortex [17]. Furthermore, a study analyzing the temporal expression of Dbx1 (supposedly specific of Cajal-Retzius cells) proposed the ventral pallium as a source of Cajal-Retzius cells [8]. However, the three tracking approaches used in the present study fail to support this hypothesis.

### Commonly used Pallial and Subpallial Markers may not be Highly Specific

The pallial marker Tbr1 [18] was expressed in almost all cells originating in the septum, which is considered a subpallial territory [19]. It is possible that tracer injection into the septal region tracks cell populations from both subpallial and pallial regions, which could explain this surprising outcome. Cells from the rostromedial telencephalic wall (RMTW), dorsal to the septum, have been shown to migrate through the septal region, and a percentage of these cells express Tbr1 [4]. Alternatively, cells generated in the septal area may express both pallial and subpallial markers. Indeed, Dbx1, a typical marker of the ventral pallium, is also expressed in the septal region (subpallium), as occurs with p73, another ventral pallium marker, which is also expressed in cells derived from both the medial and lateral pallium [6], [20]–[23].

As a part of the pallial area, all cells derived from the ventral pallium are expected to express Tbr1. However, we found this to be the case in less than 50% of the cells from this region. Although several markers are usually used when labeling cells from pallial and subpallial sources, the present findings highlight the importance of using tracers in addition to markers when ascribing origins to cells from pallial, subpallial or other regions.

### Summary

Tangential migration is the most common migratory mechanism adopted by neuroblasts that are generated in various regions of the developing brain. This process allows genetically similar cells to invade and settle in regions far from their site of origin, and it permits cells with very different genetic backgrounds to integrate into the same region. Together with our previous findings [4], the present results indicate that in early developmental stages (the preplate stage), almost all proliferative areas in the telencephalon produce cells that form part of the olfactory cortex. Accordingly, we identify the septum and ventral pallium as new sources of olfactory cortex cells, and we demonstrate that each proliferative area contributes stereotypically to a specific zone of the olfactory cortex (Fig. 5). Taken together, these findings further support the view that ontogenetic heterogeneity leads to spatial variability.

## Materials and Methods

### Animals

Fifteen C57 pregnant mice housed at the Cajal Institute were used to generate the 78 embryos analyzed in this study. The distribution of the animals used and their survival is shown in the Table 1.

In addition, we used 15 embryos from two pregnant dams of a transgenic line that expresses an enhanced green fluorescent protein (GFP) under the control of the β-actin promoter (C57BL/6-TgN [ACTbEGFP] 10sb, JAXMice). The day on which the vaginal plug was observed was defined as E0.5 and pups were born on E19.5 (P0). All animal handling and experimental protocols were in compliance with Spanish legislation (R.D. 1201/2005 and L.32/2007) and the Guidelines of the European Union Council (2003/65/CE) for the care and use of experimental animals. In addition, the animal care and use committee of the Cajal Institute approved all procedures. Pregnant dams were anesthetized with an intraperitoneal injection of Equithesin (3 ml/kg) before surgery and sacrificed by cervical dislocation. Embryos were removed by caesarean section at different developmental stages (E10.5, E11.5 and E12.5).

### Whole Embryo Culture

Whole embryos were cultured using an incubator and a roller bottle system based on Cockroft’s protocol (1990), as previously described in detail [3]. Briefly, mouse embryos were removed from the uterus and placed in a Petri dish containing Hank’s balanced solution (HBS) at 37°C in sterile conditions. The muscular uterine wall and the decidua were removed, and Reichert’s membrane was detached to reveal the vascularized visceral yolk sac containing the embryo. Taking care to maintain the integrity of the vitelline arteries and veins, the avascular side of the yolk sac was partially broken to expose the embryo. The amnion was removed and the vessels of the vitelline stalk tucked under the tail of the embryo. Tracers (in crystal form or in solution: see below) were injected (100 nl) with the aid of a dissecting microscope (Nikon SMZ1500) and a pressure device (4 pulses of 6 ms and 45 psi: Picospritzer, General Valve, Cameron, AR). Subsequently, the injected embryo was transferred to a glass bottle containing 4 ml of culture medium, which was placed in an incubator for one day at 35°C (95% O_2_, 5% CO_2_). Embryos were cultured in medium containing heat-inactivated rat serum obtained by centrifugation of blood (100 *g*×5 min, 3 times) immediately after extraction from the donor animal. This serum was filtered with Filtropur S 0.45 (Sarstedt, Nümbrecht) and supplemented with 1 mg/ml of glucose and antibiotic (penicillin-streptomycin, 100 IU/ml: Gibco, Grand Island, NY).

### Intrauterine Experiments

Embryos were injected with a fluorescent tracer in the brain structure of interest using an ultrasound guided injection system, VeVo 770® (VisualSonics Inc. Toronto, Canada) to specifically label newly generated cells, as described previously [5]. Briefly, E10.5–E11.5 pregnant mice were anesthetized with isoflurane (Isova® vet, ref. 240055: Centauro, Barcelona, Spain) and the uterine horns were exposed from the abdominal wall and covered with pre-warmed ultrasound gel (Parker Laboratories, Inc. NJ USA). Using a micromanipulator, each embryo was injected with a total volume of 100 nl of dye into the ventral pallium or septal area of the developing encephalon. Finally, the ultrasound gel was removed and the uterine horns were set back into the abdomen. After surgery, the pregnant dams were treated with the antibiotic enrofloxacin (*Baytril,* 5 mg/kg: Bayer, Leverkusen, Germany) and the anti-inflammatory ketorolac (*Droal,* 300 µg/kg: VITA Laboratories, Barcelona, Spain).

### Cortical Hem Implants

GFP-expressing transgenic and wild type (wt) mouse embryos were used for cortical hem implants. The telencephalon was removed from GFP-expressing embryos under a dissecting microscope (Nikon SMZ1500, Nikon Corp., Tokyo) using fine watchmaker’s forceps. The tissue for implantation was obtained from the caudal portion of the hem, decreasing the likelihood of extracting the surrounding mesenchyme. Using a hydraulic (oil filled) system, sections of solid tissue were removed from the caudal GFP-cortical hem and implanted into the septal area of wt embryos. The injection system consisted of a micropipette glued to a microperfusor (25G, 0.5×19 mm: Pic Indolor, Como, Italy) connected by a rubber tube to a 1 ml syringe (25 GA, 0.5×16 mm: BD Plastipak, Madrid, Spain), and filled with an interface of HBS and mineral oil.

### Tracer Injections

Two different fluorescent tracers were used: carboxy-fluorescein diacetate succinimidyl ester (CFDA SE, 10 mM solution in DMSO: 557 PM, Molecular Probes) and 1, 1′-dioctadecyl-3, 3, 3′, 3′-tetramethylindocarbocyanine perchlorate (DiI crystals: Molecular Probes). CFDA is a green tracer that becomes fluorescent when is taken by either dividing or migrating cells and its labeling is transferred to their progeny. DiI is a red lipophilic tracer that labels the plasma membrane.

### Immunohistochemistry

Immunohistochemical analysis of vibratome sections (40 µm thick) was performed using the following primary antibodies: mouse anti-reelin (MAB364 clone G10, 1∶1,000: Chemicon, Temecula, CA); rabbit anti-calbindin-D28K (CB, 1∶10,000: Swant, Bellinzona, Switzerland); rabbit anti-calretinin (CR, 1∶2,000: Swant); and rabbit anti-Tbr1 (1∶1,000: Chemicon). The following secondary antibodies were used: Alexa Fluor 568-conjugated goat anti-mouse (1∶2,000: A11004, Molecular Probes Inc., Eugene, OR) and Alexa Fluor 568-conjugated goat anti-rabbit (1∶2,000: A11011, Molecular Probes). Sections were pretreated with 0.2% phosphate buffered saline-Tween 20 (PBS-T) and blocked with 5% normal goat serum (NGS) and 0.1% bovine serum albumin (BSA) in 0.2% PBS-T. The sections were then incubated overnight at 4°C with the primary antibodies diluted in blocking solution, after which they were washed with 0.2% PBS-T and incubated with the appropriate secondary antibody for 90 min at 4°C. For reelin immunohistochemistry, sections were treated with cold citrate buffer (pH 6) for 5 min and with citrate at 90°C for 1 min. Finally, all sections were counterstained with bisbenzimide.

### Image Acquisition

Whole brain fluorescent images were acquired using a fluorescent dissecting microscope (Leica MZFL-III) and either a fluorescent microscope (Nikon Eclipse E600) equipped with a digital camera (Nikon DMX 1200F) or a spectral confocal microscope (Leica TCS 4D). All photographs were adjusted equally for contrast and brightness using Adobe Photoshop CS4 (Adobe, San Jose, CA).

### Co-localization Analysis

The percentage of cells co-labeled with different markers was quantified in serial confocal images taken every 2 µm from 40 µm thick vibratome sections using the Cell Calculator plug-in (University of Sheffield) for Image J software.
